# Shared pathogenesis in polycystic ovaries and rheumatoid arthritis: an analysis of key genes and pathways

**DOI:** 10.3389/fgene.2025.1554139

**Published:** 2025-06-23

**Authors:** Yingying Ji, Pengcheng Xia, Yan Wang, Yang Liu, Feng Wang, Fenggang Sun, Qiang Feng, Qingbin Ni, Yi Li

**Affiliations:** ^1^ Department of Clinical Laboratory Medicine, The Affiliated Taian City Central Hospital of Qingdao University, Taian, Shandong, China; ^2^ School of Basic Medical Sciences, Shandong First Medical University, Taian, Shandong, China

**Keywords:** PCOS, RA, bioinformatics analysis, core genes, immune dysregulation

## Abstract

**Objective:**

The study aims to explore the potential shared pathogenic processes between PCOS and RA through bioinformatics analysis to identify novel therapeutic targets and biomarkers for disease management.

**Methods:**

Microarray datasets for polycystic ovary and RA were obtained from the GEO database. Differential gene expression analysis identified commonly dysregulated genes in both conditions. Gene Ontology (GO) and KEGG pathway enrichment analyses were performed to understand the biological processes and pathways associated with the differentially expressed genes (DEGs). Protein interaction analysis, machine learning algorithms, and validation analyses were employed to identify core genes with potential diagnostic value. Immune cell infiltration analysis and evaluation of hypoxia and angiogenesis scores were conducted to assess the role of the core genes in immune-related disorders.

**Results:**

Microarray analysis identified differentially expressed genes (DEGs) commonly dysregulated in PCOS and RA. GO and KEGG enrichment analyses highlighted the involvement of cell death, inflammation, and redox pathways. Ten key genes were identified through protein interaction analysis, and machine learning further narrowed it down to six core genes: CSTA, DPH3, CAPZA2, GLRX, CD58, and IFIT1. The core genes were overexpressed in PCOS and RA tissues, suggesting their potential involvement in disease development. Validation analyses confirmed the diagnostic potential of these genes, especially in RA. Immune cell infiltration analysis correlated the expression of core genes with neutrophil and CD8^+^ T cell infiltration. Hypoxia and angiogenesis scores indicated the significance of these genes in immune-related disorders.

**Conclusion:**

The study unveils potential molecular links between PCOS and RA, highlighting the importance of immune dysregulation in their pathogenesis. The identified core genes offer novel therapeutic targets and potential biomarkers for disease management, providing insights into the complex interplay between these two seemingly unrelated conditions.

## 1 Introduction

Rheumatoid arthritis (RA) is a chronic autoimmune disorder with significant global impact, characterized by polyarthritis, fatigue, and systemic inflammation (Josef et al., 2016). It imposes a substantial health burden, particularly affecting women and can progress from mild symptoms to severe disability, greatly impacting an individual’s quality of life (Ennio Giulio et al., 2018). The pathogenesis of RA is closely tied to immune dysregulation, leading to autoimmunity against joint tissues and systemic inflammation affecting multiple organ systems (Stefano et al., 2022).

The higher prevalence of RA in women is believed to be influenced by hormonal factors, with hormonal imbalances potentially contributing to immune dysfunction and the development of RA (Deshiré et al., 2016; Joseph et al., 2023). Polycystic ovary syndrome (PCOS), a common hormonal disorder in women, is associated with immune system dysregulation and has been linked to an elevated risk of autoimmune diseases (Hifsa et al., 2016; Ying-Yi et al., 2022). Studies suggest a possible association between PCOS and RA, possibly due to shared genetic factors and hormonal influences impacting immune responses (Héctor, 2018; Katherine et al., 2024). Hormonal imbalances, such as those observed in PCOS, can disrupt immune function and increase susceptibility to autoimmune conditions like RA (Jingxuan et al., 2023). Metabolic abnormalities, such as insulin resistance in PCOS, have also been implicated in the pathogenesis of RA (Miguel and Manuel, 2020). Genetic links between RA and PCOS have been established, emphasizing the importance of further investigations to identify diagnostic markers and therapeutic targets for RA in the context of PCOS. Utilizing advanced technologies like RNA sequencing and microarray analyses can offer valuable insights into the molecular mechanisms underlying the association between PCOS and RA (Shuji et al., 2018; Zanjirband et al., 2023). By identifying differentially expressed genes and pathways associated with both conditions, researchers can uncover potential commonalities and novel targets for diagnostic and therapeutic advancements.

Further research is warranted to deepen our understanding of the genetic and molecular connections between PCOS and RA. The present study aims to investigate shared genetic elements between these disorders using bioinformatics analyses of gene expression datasets to elucidate core genes and pathways involved in their pathogenesis. The subsequent sections will detail the results and implications of these analyses, shedding light on the potential interplay between PCOS and RA for improved disease management strategies.

## 2 Materials and methods

### 2.1 Microarray datasets acquisition

Microarray datasets from polycystic ovaries and rheumatoid arthritis were retrieved from the GEO database (https://www.ncbi.nlm.nih.gov/geo/) with the accession numbers GSE54250 (8 healthy individuals and 8 with PCOS) and GSE93272 (43 healthy controls and 232 with RA). Since PCOS is a female-specific disease, only females from the two datasets were included in this study. The data underwent normalization and log transformation to address potential skewness issues. Table 1 shows more information on these datasets.

**TABLE 1:** The summary of datasets used in this study.

GEO accession	Platform	Tissue type	Samples
GSE54250	GPL10558	peripheral blood samples in PCOS patients	8 vs. 8
GSE93272	GPL570	Whole blood gene expression of RA	43 vs. 232

Notes: Annotation: (Illumina HumanHT-12 V4.0 expression beadchip); GPL570: (hgu133plus2).

### 2.2 Data preprocessing

Data analysis was conducted using the R software package (version 4.4.2; https://www.r-project.org/). Probes lacking annotation were excluded, and duplicate expression values were averaged. Differentially expressed genes (DEGs) were identified utilizing the limma package (Matthew et al., 2015) from Bioconductor (https://www.bioconductor.org/), applying screening criteria of P < 0.05 and |logFC| > 0.6.

### 2.3 Co-expressed gene extraction and screening

Co-expressed genes in the two datasets were extracted and intersecting genes were screened as potential key genes using Venn diagrams (Philippe et al., 2014).

### 2.4 GO and KEGG enrichment analyses

To analyze the biological functions and pathways, Gene Ontology (GO) enrichment and Kyoto Encyclopedia of Genes and Genomes (KEGG) pathway enrichment analyses were performed based on the common differentially expressed genes using the R package clusterProfiler (Guangchuang et al., 2012).

### 2.5 Protein-protein interaction (PPI) network

A protein-protein interaction (PPI) network of the DEGs was built using the STRING database (https://string-db.org/) and visualized using Cytoscape software (https://cytoscape.org/). The CytoHubba plug-in in Cytoscape was used to select pivotal genes from the PPI network based on eight topological approaches (Chia-Hao et al., 2014).

### 2.6 Machine learning

Machine learning was carried out using an online web-based tool available at http://owo-b.com/index. A random forest algorithm, an ensemble machine learning method, was used to screen the core genes in GSE54250 and GSE93272. The intersection of these genes signifies core targets for polycystic ovaries and rheumatoid arthritis.

### 2.7 Verification of pivotal genes

The reliability of the identified genes was verified in a dataset containing samples related to polycystic ovary (GSE54250) and rheumatoid arthritis (GSE93272). The expression of these genes was first verified and compared with normal controls. To evaluate the predictive efficiency for disease of the identified critical genes, the accuracy of hub genes was evaluated by ROC validation and the area under the curve (AUC) values were calculated by an online website (https://www.xiantao.love/products/). Efficacy evaluation: non-efficiency (AUC ≤ 0.5); modest-efficiency (0.5 < AUC < 0.7); high-efficiency (AUC > 0.7).

### 2.8 Validation of the expression of the pivotal genes

Blood samples were obtained from 30 healthy controls, 30 patients with PCOS, and 30 patients with RA at the Tai’an Central Hospital affiliated Qingdao University between 2023 and 2024. All participants provided informed consent and the study adhered to the ethical requirements of the Helsinki Declaration. Ethical approval was obtained from the Ethics Committee of the Tai’an Central Hospital affiliated Qingdao University (2023-06-35). RNA was extracted from peripheral blood using TRIzol reagent (Invitrogen, United States) and cDNA was synthesized using a reverse transcription kit (Applied Biosystems) according to the manufacturer’s instructions. Quantitative real-time PCR analysis was performed using the KAPA SYBR Green Fast BioRad icycler kit (Peqlab) on a BioRad CFX96 real-time PCR system. The expression levels of target genes were calculated relative to β-actin using the 2^−ΔΔCt^ method. The primer sequences are as follows (Table 2).

**TABLE 2:** Primer sequence for qRT-PCR.

Target gene		Primer sequence (5′–3′)
CSTA	Forward	5′-TCC​AGA​AAT​CCA​GGA​GAT​TGT​TGA-3′
	Reverse	5′-ATC​ACC​TGC​TCG​TAC​CTT​AAT​GT-3′
DPH3	Forward	5′-CGA​GGA​CTT​CCA​ATA​TGA​CGA​GG-3′
	Reverse	5′-AAT​GAG​AGA​GCA​GCT​AGG​ACA​C-3′
CAPZA2	Forward	5′-TGG​AGT​CTG​CAC​TGT​GTA​TGG-3′
	Reverse	5′-TGA​GTG​GTT​GAA​GGA​GTG​ATT​GT-3′
GLRX	Forward	5′-CAC​AGC​CAC​CAA​CCA​CAC​TAA​C-3′
	Reverse	5′-GAC​TAG​ATC​ACT​GCA​TCC​GCC​TAT-3′
CD58	Forward	5′-CTG​TGT​CAG​GTA​GCC​TCA​CTA​TC-3′
	Reverse	5′-TGC​ACA​AGT​TAG​TGT​GGG​AGA-3′
IFIT1	Forward	5′-GCC​TCC​TTG​GGT​TCG​TCT​ACA-3′
	Reverse	5′-GGA​CCT​TGT​CTC​ACA​GAG​TTC​TCA-3′
BACTIN	Forward	5′-GGC​ACC​CAG​CAC​AAT​GAA​G-3′
	Reverse	5′-CCG​ATC​CAC​ACG​GAG​TAC​TTG-3′

### 2.9 Construction of miRNA-mRNA network

Genes were predicted to be targeted by miRNAs in miRDB (https://mirdb.org/). A network of miRNA-mRNA was constructed using Cytoscape software.

### 2.10 Correlation analysis

Correlation analysis between hub gene expression and the number of neutrophils was conducted. Also, the correlation between hub genes and immune cells were analyzed using Pearson correlation coefficient.

### 2.11 CIBERSORTs analysis

Analysis of GSE54250 and GSE93272 was conducted using CIBERSORTs (Aaron et al., 2015) (https://cibersort.stanford.edu/) to evaluate the difference in immune cell types between the two conditions.

### 2.12 Hypoxia and angiogenesis scores

Hypoxia scores and angiogenesis scores were calculated using the ssGSEA method as described in the study (Li et al., 2009) which are critical for immune-related diseases, were calculated. Correlation analysis was conducted to assess the relationship between these scores and the expression of the identified genes.

### 2.13 Statistical analyses

R studio software (Version 4.2.3) and GraphPad Prism 8.0 software were used to draw graphics and conduct statistical analysis. All of the data are shown as mean ± SD. *P* < 0.05 indicated statistical significance.

## 3 Results

### 3.1 Data acquisition and initial analysis

**FIGURE 1:**
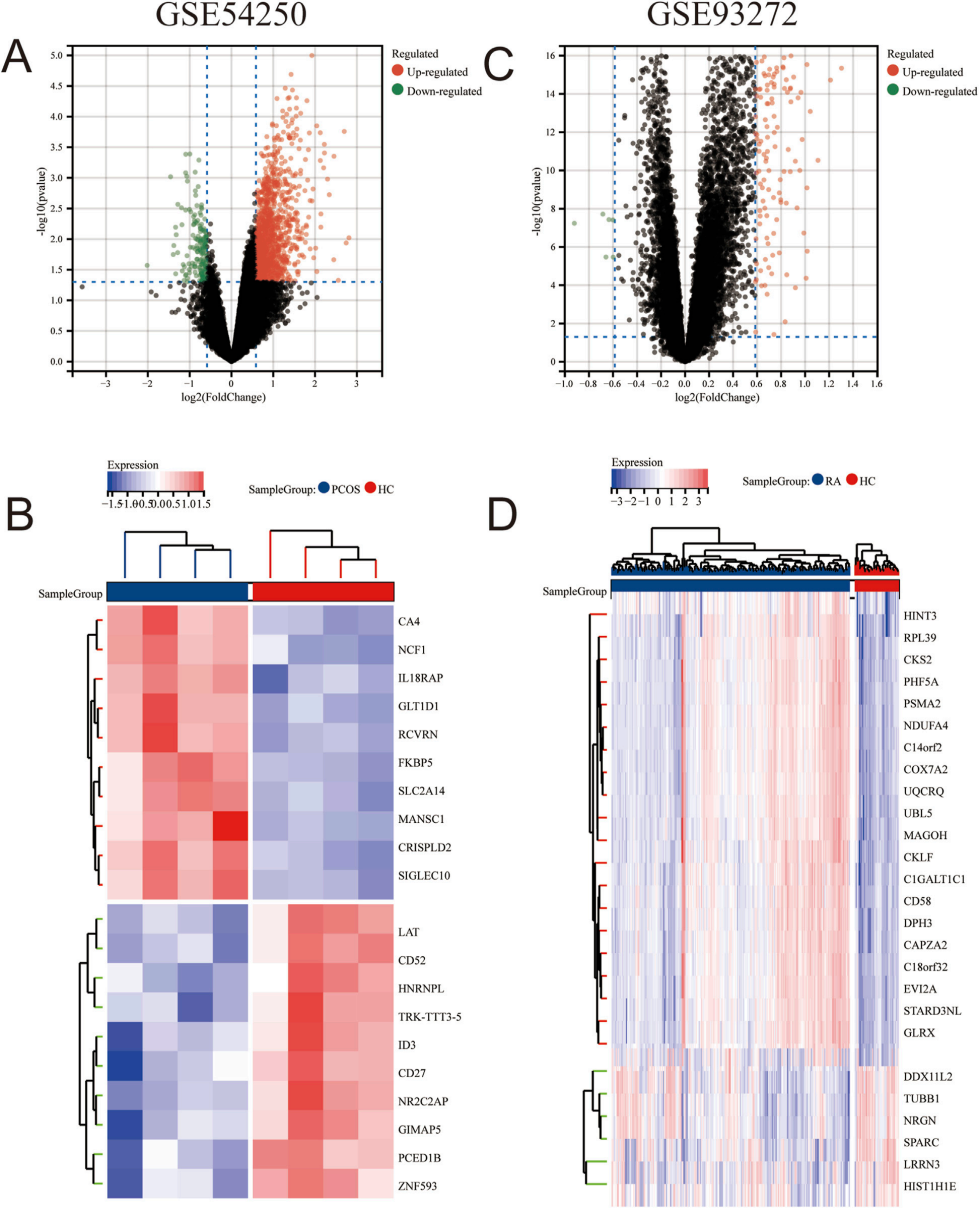
Differential Gene Analysis between PCOS and RA Gene Sets **(B)** Volcano plot of GSE54250. **(B)** Heatmap of differentially expressed genes in GSE54250. **(C)** Volcano plot of GSE93272. **(D)** Heatmap of differentially expressed genes in GSE93272.

To identify genes co-expressed in polycystic ovaries and rheumatoid arthritis, we obtained microarray data from two datasets (GSE54250 and GSE93272) as a training set. After normalization and log-transforming the data, we used the R software to remove probes without annotation information and calculated averages in the presence of duplicate expression data. Genes with screening criteria of *P* < 0.05 and |logFC|>0.6 were identified as differentially expressed genes (DEGs). A total of 425 DEGs (396 upregulated and 29 downregulated genes) were identified in the GSE54250 dataset and 182 DEGs (176 upregulated and 6 downregulated genes) were identified in the GSE93272 dataset. Differential genes were displayed as volcano plots in Figures 1A,C, respectively, and the top-ranked differential genes were displayed with clustered heatmaps (Figures 1B,D).

### 3.2 Identification of Co-expressed genes and pathway analysis

**FIGURE 2:**
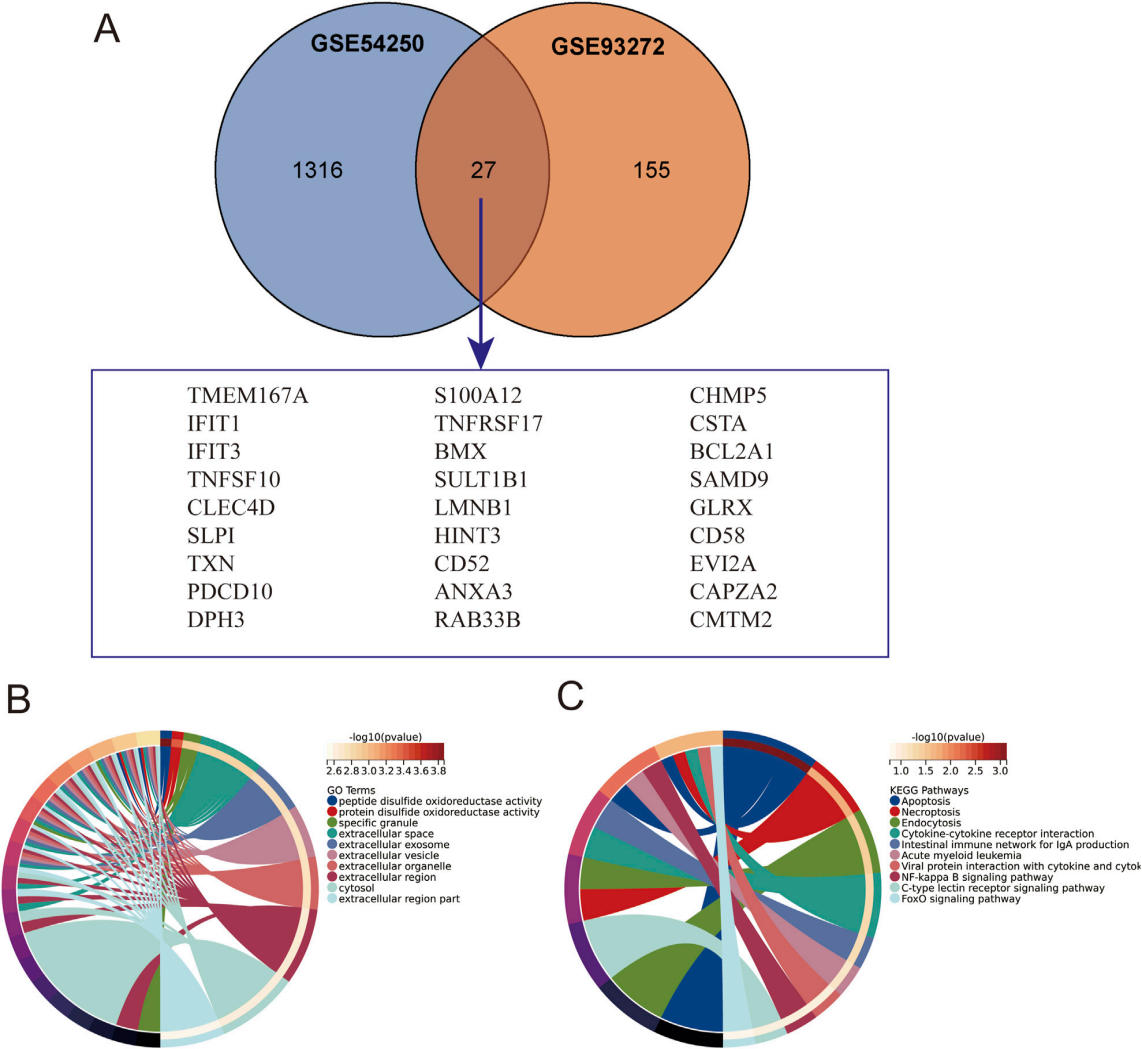
Intersection of Gene Sets and Enrichment Analysis. **(A)** Intersection of GSE54250 and GSE93272 gene sets. **(B)** Gene Ontology (GO) enrichment analysis of the intersection genes. **(C)** Kyoto Encyclopedia of Genes and Genomes (KEGG) pathway enrichment analysis of the intersection genes.

Subsequently, we extracted the co-expressed genes in the two datasets and screened 27 intersecting genes as potential key genes by Venn diagrams (Figure 2A), suggesting that polycystic ovaries and rheumatoid arthritis may share a common pathogenesis. To further analyze the biological functions and pathways, we performed GO enrichment and KEGG pathway enrichment analyses based on the common differentially expressed genes. As shown in Figures 2B,C, the results of KEGG enrichment analysis showed that the apoptosis, necroptosis, and endocytosis pathways were significantly enriched; The GO enrichment results showed that the differences were mainly in peptide disulfide oxidoreductase activity, protein disulfide oxidoreductase activity, and specific granule. Taken together, these results strongly suggest that the intracellular environment and immune cell function play an important role in the pathogenesis of polycystic ovaries and rheumatoid arthritis.

### 3.3 Protein-protein interaction network and core gene identification

**FIGURE 3:**
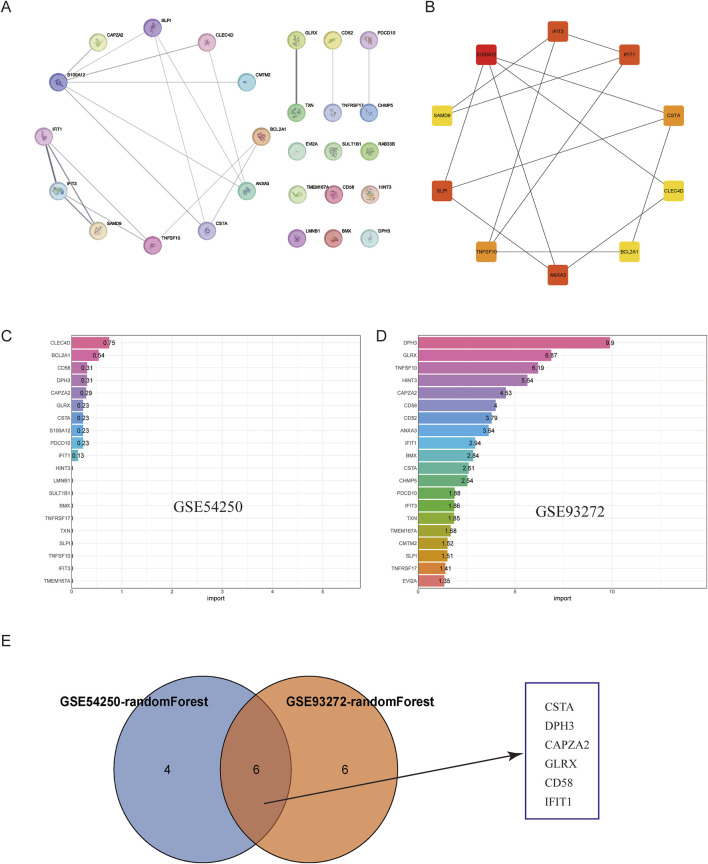
Construction of PPI Network and Machine Learning Selection. **(A)** Protein-Protein Interaction (PPI) network of the intersection genes. **(B)** Hub genes identified in the PPI network. **(C)** Random forest selection of key genes from GSE54250. **(D)** Random forest selection of key genes from GSE93272. **(E)** Intersection of selected genes using Venn diagram.

To further investigate the interactions of common differentially expressed genes (DEGs) between polycystic ovaries and rheumatoid arthritis, we built a protein-protein interaction (PPI) network of 27 DEGs based on the STRING database and visualized it using Cytoscape software (Figure 3). We used the CytoHubba plug-in in Cytoscape to select pivotal genes from the PPI network based on eight topological approaches including intermediacy, bottleneck, EPC, degree, MCC, MNC, radiality, and stress (Table 1). After cross-tabulation analysis, we identified 10 genes: S100A12, IFIT1, IFIT3, ANXA3, SLPI, TNFSF10, CSTA, CLEC4D, SAMD9, and BCL2A1. And obtained the functional information of these genes (Table 2). Therefore, a random forest algorithm in machine learning was used to screen the core genes in GSE54250 and GSE93272 to take the intersection and obtain CSTA, DPH3, CAPZA2, GLRX, CD58, IFIT1. These genes can be considered as the core targets for polycystic ovaries and rheumatoid arthritis.

### 3.4 Gene expression verification and diagnostic ability evaluation

**FIGURE 4:**
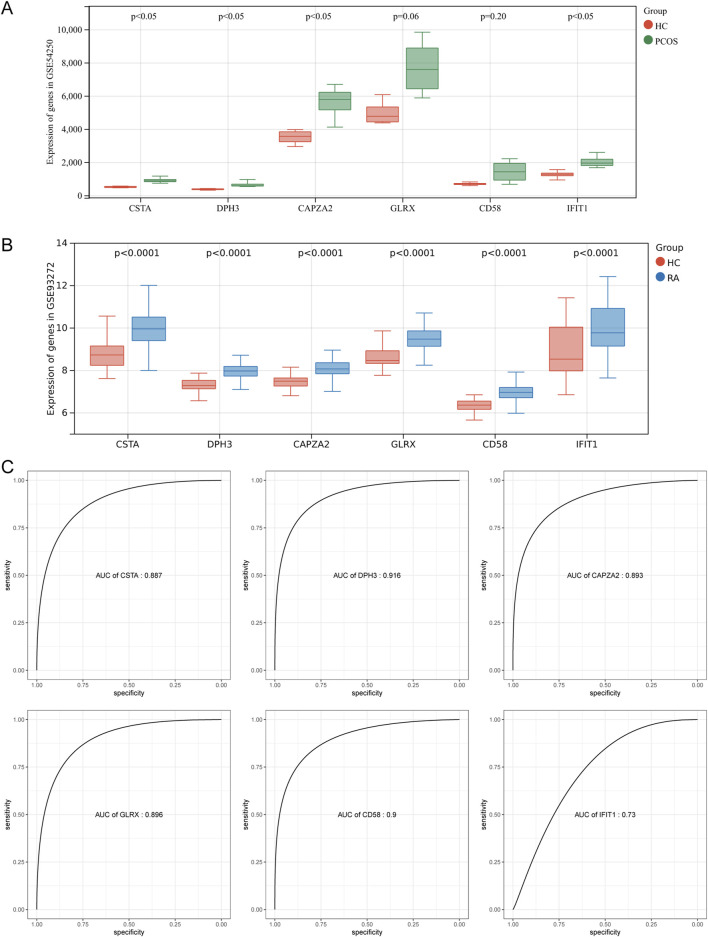
Validation of Hub Genes and ROC Efficiency. **(A)** Validation of hub genes in PCOS. **(B)** Validation of hub genes in RA. **(C)** ROC curve analysis for diagnostic efficiency of hub genes in RA.

To verify the reliability of these six pivotal genes, the expression of these genes was first verified in a dataset containing samples related to polycystic ovary GSE54250 and rheumatoid arthritis GSE93272. As shown in Figures 4A,B, four genes were significantly upregulated in polycystic ovary and all six genes were significantly upregulated in rheumatoid arthritis compared with normal controls. To further evaluate the diagnostic ability of the hub genes in the two diseases, we plotted ROC curves based on these gene expression data. Due to the small number of samples in the PCOS group, it was not meaningful to plot ROC curves; therefore, ROC curves were plotted only for the expression data in GSE93272. The AUCs were 0.887 for CSTA, 0.916 for DPH3, 0.893 for CAPZA2, 0.896 for GLRX, 0.90 for CD58, and 0.73 for IFIT1 (Figure 4C). Taken together, these pivotal genes may have a strong ability to differentiate between polycystic ovaries and rheumatoid arthritis as potential biomarkers.

### 3.5 GSEA analysis and immune cell analysis

**FIGURE 5:**
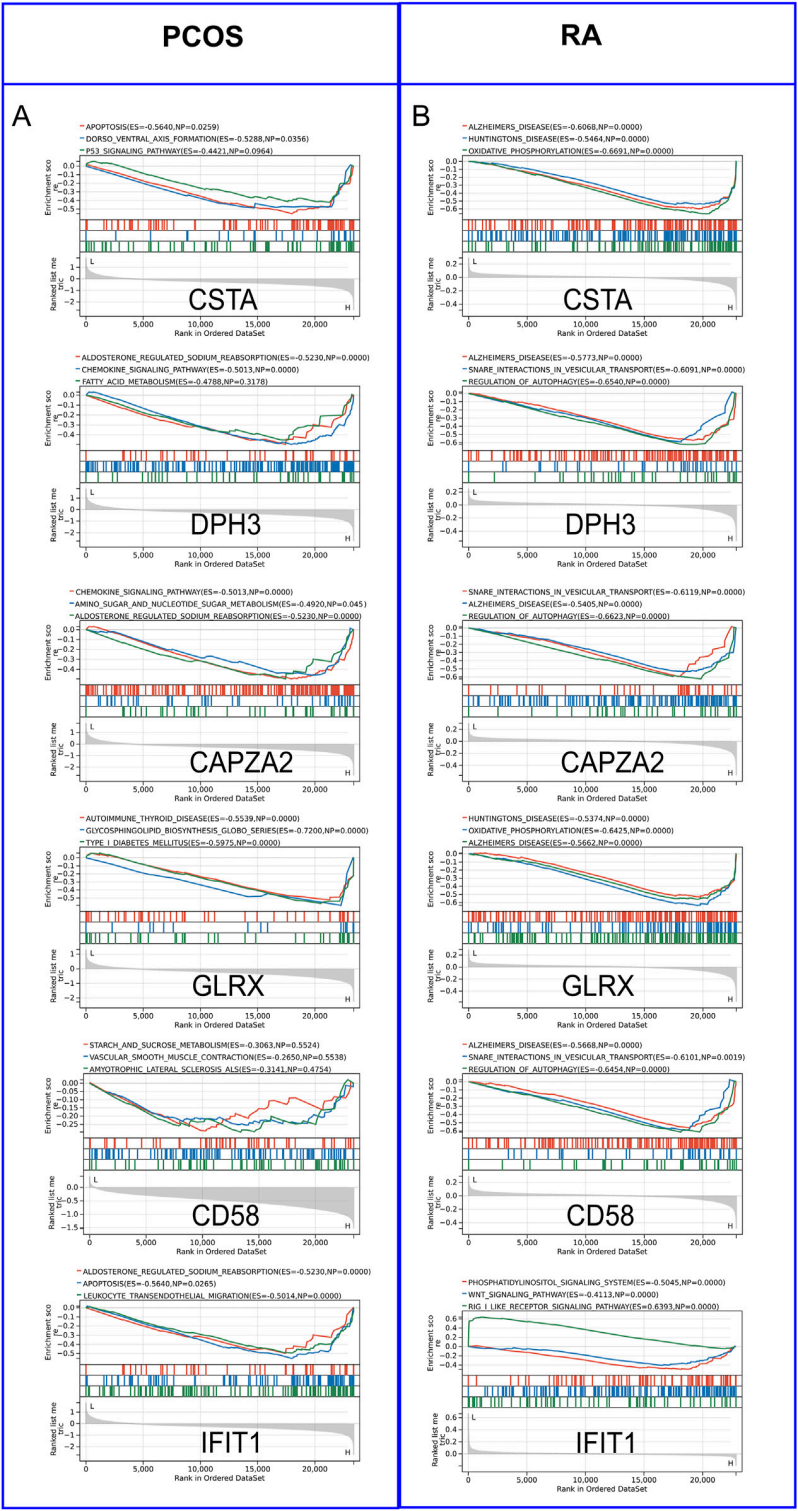
Gene Set Enrichment Analysis (GSEA) of Hub Genes. **(A)** GSEA analysis of hub genes in PCOS. **(B)** GSEA analysis of hub genes in RA.

**FIGURE 6:**
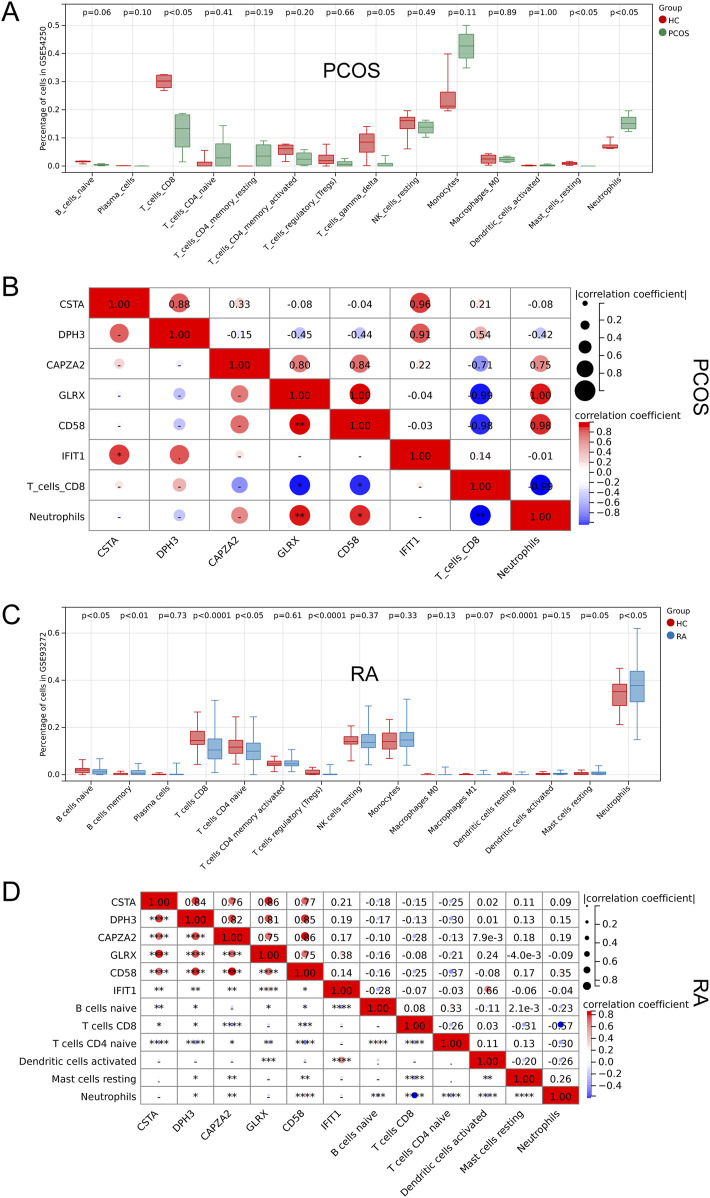
Correlation Analysis of Hub Genes with Immune Cells. **(A)** Comparison of immune cell profiles in PCOS. **(B)** Correlation between hub genes and immune cells in PCOS. **(C)** Comparison of immune cell profiles in RA. **(D)** Correlation between hub genes and immune cells in RA.

By performing GSEA analysis of six Hub gene in PCOS and RA, respectively, it was observed that the KEGG pathways that function in PCOS (Figure 5A) and RA (Figure 5B) are different. Analysis of GSE54250 and GSE93272 by CIBERSORTs revealed significant differences in neutrophils and CD8^+^ T cells, among others, in GSE54250 (Figure 6A); and in GSE93272, neutrophils and CD8^+^ T cells, among others, also differed significantly (Figure 6B). Subsequently, by correlation analysis of hub genes and immune cells, it was found that in polycystic ovaries, GLRX was positively correlated with neutrophils (R = 1.00) and negatively correlated with CD8^+^ T cells (R = −0.99), and that CD58 was positively correlated with neutrophils (R = 0.98) and negatively correlated with CD8^+^ T cells (R = −0.98) (Figure 6C); in RA, IFIT1 was positively correlated with DC cells (R = 0.66) (Figure 6D).

### 3.6 Hypoxia and angiogenesis scores analysis

**FIGURE 7:**
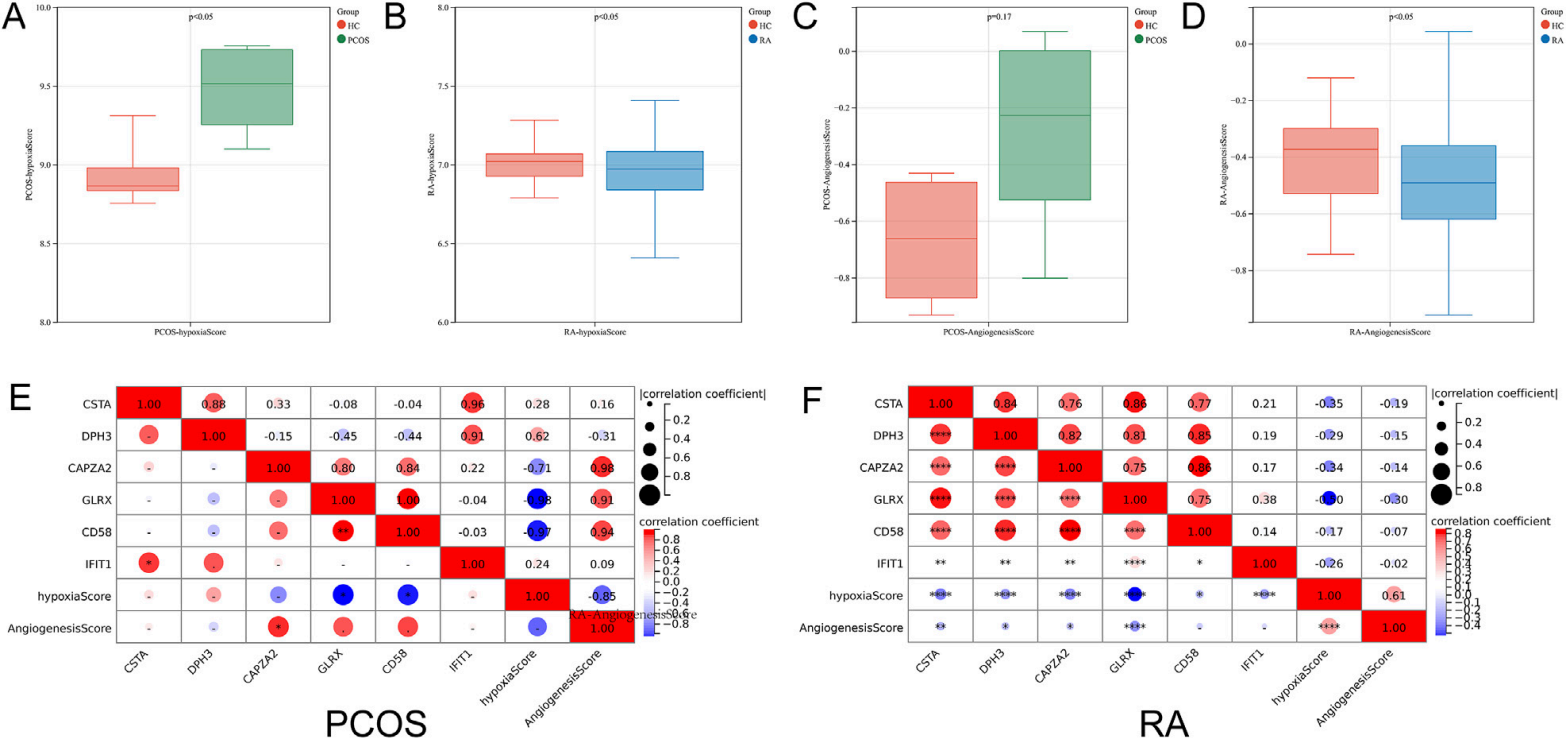
Correlation Analysis of Hub Genes with Hypoxia and Angiogenesis Scores. **(A)** Comparison of hypoxia scores in PCOS. **(B)** Comparison of hypoxia scores in RA. **(C)** Comparison of angiogenesis scores in PCOS. **(D)** Comparison of angiogenesis scores in RA. **(E)** Correlation between hub genes and hypoxia scores in PCOS. **(F)** Correlation between hub genes and angiogenesis scores in RA.

Hypoxia scores and angiogenesis scores are critical for immune-related diseases, and the analysis revealed significant differences in hypoxia scores in PCOS and in both hypoxia scores and angiogenesis scores in RA (Figures 7A-D). Correlation analysis revealed that GLRX and CD58 were negatively correlated with hypoxia score in PCOS, and all six hub genes were negatively correlated with hypoxia score in RA, and CSTA, DPH3, CAPZA2 and GLRX were negatively correlated with angiogenesis score (Figures 7E,F).

### 3.7 Blood sample validation and miRNA-mRNA network

**FIGURE 8:**
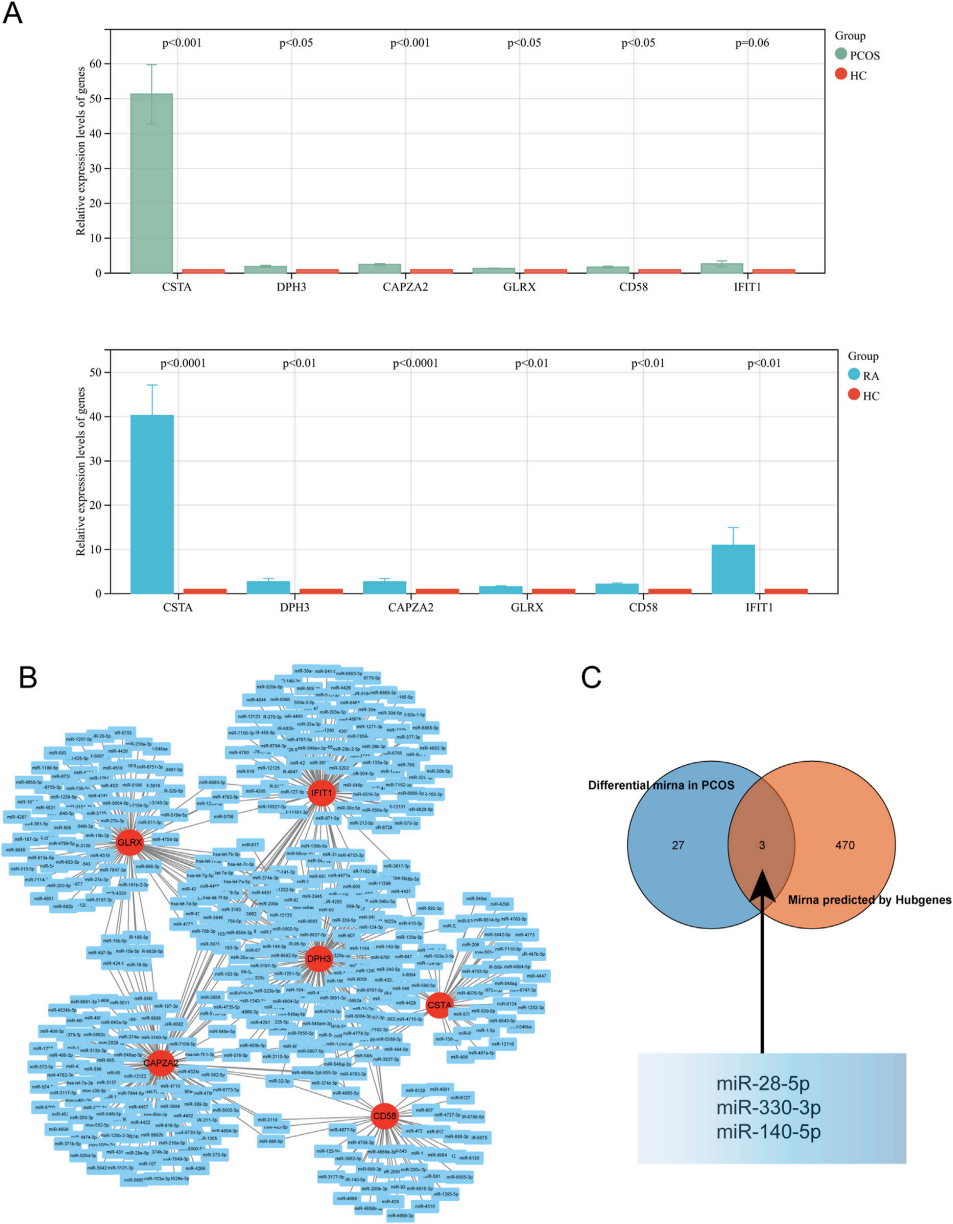
Validation and Network Analysis of Hub Genes. **(A)** Expression levels of hub genes in the validation cohort for PCOS and RA. **(B)** miRNA network associated with hub genes. **(C)** Intersection of predicted miRNA network with the dataset.

Validation of the expression levels of key genes in the collected blood samples was consistent with the analyses (Figure 8A); A miRNA-mRNA network was constructed using Cytoscape (Figure 8B); among the miRNAs predicted by the key genes, there were three intersections with differential miRNAs: miR-28-5p, miR-330-3p and miR-140-5p (Figure 8C).

## 4 Discussion

This study aims to explore the potential shared pathophysiological mechanisms between polycystic ovary syndrome (PCOS) and rheumatoid arthritis (RA), focusing on co-expressed genes and their potential as biomarkers and therapeutic targets. By analyzing microarray data from two independent datasets (GSE54250 and GSE93272), we found interesting correlations between these two diseases. Our analysis revealed 27 differentially expressed genes common to both diseases, which are associated with alterations in the intracellular environment and immune cell functions. Further analysis using the Random Forest algorithm identified six core genes with the highest potential: CSTA, DPH3, CAPZA2, GLRX, CD58, and IFIT1. Immune cell-related analysis unveiled the importance of neutrophils and CD8^+^ T cells in both diseases, particularly highlighting the strong association of GLRX, CD58, and IFIT1 with these cell types. Additionally, significant differences were observed in hypoxia and angiogenesis scores between patients with PCOS and RA, suggesting that microenvironmental regulation in immune-related diseases may be influenced by these critical genes. The validation phase confirmed the differential expression of these key genes and identified three miRNAs associated with them (miR-28-5p, miR-330-3p, and miR-140-5p), indicating their potential role in disease regulation.

Our study identified 27 genes that exhibited differential expression in both PCOS and RA. GO functional and KEGG pathway enrichment analyses provided further insights, suggesting that alterations in the intracellular environment and immune cell functions could be key shared factors between the two diseases. PCOS is characterized by a microenvironment within the ovary influenced by various interrelated factors, including insulin resistance, inflammation, and abnormal steroid hormone synthesis. These factors lead to hyperandrogenism, disrupting the internal environment balance within the ovary (Han et al., 2023). Additionally, the endometrium of PCOS patients displays abnormal immune characteristics, with an increased proportion of specific immune cell populations (such as macrophages) and upregulated expression of immune markers (Elisabet et al., 2024). In the case of RA, the synovial microenvironment influences the behavior of mast cells. Degranulation of mast cells in inflamed joint tissues is mediated by the MRGPRX2 receptor, a potential therapeutic target (Yunxuan et al., 2024). Recent studies have proposed nanomotor strategies to actively modulate the arthritic microenvironment, which may play a crucial role in exacerbating inflammation (Cong et al., 2022). Immune cells are pivotal in the pathogenesis of RA, impacting joint inflammation and destruction through intricate interactions and signaling pathways. Both T cells and B cells are involved in the inflammatory response, with autoantibodies produced by B cells aggravating the disease process (Noriko and Hiroshi, 2022).

By constructing a protein-protein interaction (PPI) network, we identified 10 key genes that play central roles in the network and may significantly influence the disease process. Further analysis using the Random Forest algorithm helped us identify six core genes with the highest potential: CSTA, DPH3, CAPZA2, GLRX, CD58, and IFIT1. These genes are associated with various diseases, including skin disorders, translation-related diseases, cancer, neurodegenerative diseases, and autoimmune conditions (Diamond and Farzan, 2013; Nils et al., 2014; Vikas et al., 2018; Yalu et al., 2021; Rui et al., 2022; Xiao-Man et al., 2024). CSTA inhibits proteases, protecting the skin and mucous membranes (Zhi-Jie et al., 2023). DPH3 is involved in regulating protein synthesis (Shihui et al., 2006). CAPZA2 modulates the cytoskeleton, which is essential for cell morphology and movement (Vina et al., 2023). GLRX is an antioxidant enzyme that protects cells from oxidative stress-induced damage (Qiu et al., 2021). CD58 molecules facilitate immune cell interactions and T cell activation (Zhu et al., 2006). IFIT1 participates in antiviral defense responses (McDougal et al., 2024). ROC curve analysis indicated that these genes have diagnostic value in distinguishing PCOS from RA, underscoring their potential as biomarkers.

Immune cell-related analysis revealed that neutrophils and CD8^+^ T cells play crucial roles in both PCOS and RA. Regarding neutrophils, studies have shown that they play a critical role in immune regulation in PCOS patients. Certain proteins associated with neutrophils, such as neutrophil gelatinase-associated lipocalin (NGAL) and its complex with matrix metalloproteinase-9 (MMP-9), are significantly decreased in concentration in PCOS patients, suggesting a potential protective mechanism (Ying-Yi et al., 2022). Additionally, changes in white blood cell counts and the neutrophil-lymphocyte ratio (NLR) in PCOS patients are associated with low-grade inflammation and correlate with assisted reproductive outcomes (Xin et al., 2023). In the context of CD8^+^ T cells, alterations in their function and quantity in PCOS patients are believed to be linked to the immune mechanisms of the disease (Mengting et al., 2021). Some studies indicate a reduced proportion of CD8^+^ T cells in PCOS patients, suggesting a possible disruption in their immune surveillance function (Buckner, 2010). Moreover, the functionality of CD8^+^ T cells may also be altered in PCOS patients, implying that their immune responses might differ from the norm, impacting disease progression (Cong et al., 2020). Concerning the relationship between neutrophils and RA, neutrophils play a pivotal role in the pathogenesis of RA by releasing cytotoxic molecules and exerting immunomodulatory functions (Liam and Mariana, 2019). They contribute to joint tissue damage and interact with fibroblasts in the synovium, inducing antigen-presenting functions and an inflammatory phenotype (Lucy-Jayne et al., 2021). Additionally, extracellular traps (NETs) released by neutrophils are implicated in the pathogenesis of RA, leading to a loss of immune tolerance in patients and the production of autoantibodies (Venizelos, 2017). CD8^+^ T cells are also critical in RA, and their homeostatic alterations are associated with the onset and persistence of the disease. These cells exhibit a heterogeneous phenotype, including both pro-inflammatory and anti-inflammatory characteristics, and they lose their sensitivity to regulation in the chronic inflammatory environment (Alessandra and Femke, 2016). Newly identified tissue-resident populations of CD8^+^ T cells and their interactions with antigen-presenting cells may play pivotal roles in the pathology of RA (Justin et al., 2020).

Notably, GLRX, CD58, and IFIT1 exhibit significant associations with these immune cell types. GLRX, or glutaredoxin, is a redox regulator that plays a crucial role in immune responses (Fernando et al., 2021). Neutrophils produce a substantial amount of reactive oxygen species (ROS) during their functions (Winterbourn et al., 2016), and GLRX, with its antioxidant properties, helps maintain redox balance, thereby protecting cells from damage. Studies have shown that GLRX is highly enriched in certain tumors and is closely associated with tumor immune processes (Yuanhao et al., 2020). CD58, also known as Lymphocyte Function-Associated Antigen-3 (LFA-3), is a cell surface protein that plays a critical role in immune responses and the regulation of intercellular interactions in the immune system (Makgoba et al., 1989). Its expression on neutrophils and CD8^+^ T cells is closely linked to immune functions and regulation. CD58 interacts with CD2 on the surface of neutrophils, contributing to the regulation of T cell activation and cytokine production, and it can enhance neutrophil survival and antimicrobial capacity (Rölle et al., 2016). In the context of CD8^+^ T cells, CD58 interaction with CD2 on antigen-presenting cells or target cells is essential for effective T cell activation and function, facilitating immunological synapse formation and antigen presentation (Leitner et al., 2015). IFIT1, also known as Interferon-Induced Protein with Tetratricopeptide Repeats 1, is an important component of the innate immune response and exhibits specific relationships with neutrophils and CD8^+^ T cells (Wu et al., 2023). IFIT1 expression in neutrophils enhances their antimicrobial capacity, particularly against viral infections (Diamond and Farzan, 2013). In CD8^+^ T cells, IFIT1 expression can bolster their antiviral functions and cytotoxicity by inhibiting viral replication (Franco et al., 2023).

Additionally, we analyzed hypoxia and angiogenesis scores, as they are closely related to immune-associated diseases (Carmeliet, 2003; Luo et al., 2022). The results showed significant differences in these scores between PCOS and RA patients. The analysis underscored the correlation between key genes and these scores. For instance, in PCOS, GLRX and CD58 were negatively correlated with hypoxia scores, suggesting their potential involvement in regulating oxygen partial pressure within the microenvironment. In RA, all six core genes were negatively correlated with hypoxia scores, indicating a broader role for these genes in modulating hypoxia in this autoimmune disease. Furthermore, CSTA, DPH3, CAPZA2, and GLRX were negatively correlated with angiogenesis scores in RA patients, implying that these genes may play a pivotal role in the disease’s characteristic angiogenic processes.

However, our study has some limitations. Firstly, our findings are based on bioinformatics analyses and require experimental validation to confirm the results. Additionally, we did not consider the potential impact of demographic factors such as age, ethnicity, and lifestyle, which may affect the universality of the results. Future studies should aim to validate the findings in different patient populations and utilize experimental models to elucidate the exact roles of the identified genes in the pathogenesis of the diseases.

## 5 Conclusion

In conclusion, this study provides novel insights into the shared molecular mechanisms underlying PCOS and RA, offering potential avenues for therapeutic development and disease management strategies. Future research should endeavor to refine our understanding of these shared pathways and their role in disease pathogenesis for improved patient outcomes.

## Data Availability

Publicly available datasets were analyzed in this study. This data can be found here: https://www.ncbi.nlm.nih.gov/geo/query/acc.cgi?acc=GSE54250 https://www.ncbi.nlm.nih.gov/geo/query/acc.cgi?acc=GSE93272.
